# Drug information-seeking behaviour among Jordanian physicians: a cross-sectional study

**DOI:** 10.3389/fphar.2023.1264794

**Published:** 2023-11-13

**Authors:** Sura Al Zoubi, Lobna Gharaibeh, Enas A. Amaireh, Ghaidaa S. Khlaifat, Haya M. Diab Khalayla, Sajedah N. Obeid, Khaled A. Abukhalaf, Amer M. AlSalamat, Zaha Al-Zoubi

**Affiliations:** ^1^ Department of Basic Medical Sciences, School of Medicine, Al-Balqa Applied University, As-Salt, Jordan; ^2^ Pharmacological and Diagnostic Research Center, Biopharmaceutics and Clinical Pharmacy Department, Faculty of Pharmacy, Al-Ahliyya Amman University, Amman, Jordan; ^3^ Al Hussain Al-Salt New Hospital, As-Salt, Jordan; ^4^ Jordan University Hospital, Amman, Jordan; ^5^ School of Medicine, Al-Balqa Applied University, As-Salt, Jordan; ^6^ Department of Obstetrics and Gynecology, Jordanian Royal Medical Services, Amman, Jordan; ^7^ Independent Researcher, Amman, Jordan

**Keywords:** information-seeking behaviour, drug information, information sources, challenges, Jordan

## Abstract

**Background:** Due to the huge number of drugs available and the rapid growth and change in drug information, healthcare professionals, especially physicians, frequently require reliable, easily accessible, rapid, and accurate reference sources to obtain the necessary drug information. Several sources of information are available for physicians to use and select from; however, the information-seeking behaviour of healthcare providers is varied, and this process can be challenging.

**Objectives:** In this study, Jordanian physicians were approached to evaluate the drug information they require, the sources of information they use, the perceived credibility of the sources, and the challenges they face when searching for the most accurate and current information about drugs.

**Methods:** This is an observational, cross-sectional study. A self-administered questionnaire was distributed to practising physicians in Jordan using a convenience sampling method (purposive sampling followed by snowball sampling) regardless of their speciality, age, gender, seniority, or place of employment.

**Results:** Three hundred and eighty physicians participated in the study. Most participants responded that they performed drug information searches on a weekly (155, 40.8%) or a daily basis (150, 39.5%). The drug-related information that physicians most frequently searched for concerned dosage regimens and adverse drug events. The majority of surveyed doctors (97.9%) reported using online websites to acquire drug information; UpToDate^®^, Medscape and Drugs.com were the most frequently used online databases, although many participants did not consider online sources to be the most reliable source. The most prevalent and recurrent challenges encountered concerned an inability to access subscription-only journals and websites (56.6%), difficulty identifying trusted and credible sources (41.8%) and the enormous number of available sources (35.3%). However, these challenges were less of a problem for physicians who currently work or have previously worked in academia (*p* < 0.001).

**Conclusion:** This study demonstrated that Jordanian physicians frequently use online websites to look for drug information and all doctors face challenges throughout this process particularly those with no experience in academia. This suggests that being in academia makes the process of information-seeking easier which highlights the need for academics to transfer their knowledge and experience to their non-academic colleagues and the upcoming generations of physicians.

## 1 Introduction

Prescribing drugs is a vital component in the complex process of drug utilisation and is a common practice among healthcare providers, mainly physicians, for the management of numerous health conditions via treatment or prevention (Maxwell, 2016). Possessing appropriate and up-to-date knowledge about drugs is crucial for rational drug use and optimal patient outcomes (Ely et al., 2002; Mohamadloo et al., 2017) in terms of safety, efficacy and cost (World Health Organization, 2002) by treating the correct patient with the required drug, dosage, route of administration and duration (Mehta and Gogtay, 2005). Using drugs rationally helps to avoid, or at least minimise, preventable adverse drug events (ADEs), medication errors (MEs) and unnecessary healthcare expenditure (Kar et al., 2010; Mekonnen et al., 2021). However, this can be challenging due to constant change and the rapid growth of drug information. Currently, more than 20,000 FDA-approved prescription drugs are available on the market with an average of forty-three more drugs approved annually by the FDA’s Centre for Drug Evaluation and Research (FDA, 2021; FDA, 2023).

This extensive range of drugs makes it impossible for prescribers to know and recall all of the necessary drug information such as their approved indications, dosage regimens, interactions, mechanisms of action and price at the point of care. Therefore, physicians and other healthcare professionals regularly require access to reliable, easily accessible, prompt, and accurate sources of drug information to keep them informed about the most recent drug updates and their use in treatment (Dawes and Sampson, 2003).

Information-seeking behaviour varies among healthcare providers and several sources of drug information are available to use and to choose from (Dawes and Sampson, 2003). Despite being current and providing precise information, acquiring information from primary resources such as clinical studies and reports can be challenging, especially for junior doctors, as it is a time-consuming process that requires advanced interpretation skills. Additionally, access to some studies may be limited by the subscription policies of some medical journals which further complicates the process (Shields and Park, 2019). Several studies demonstrate that physicians, and other healthcare providers, prefer to use faster, easily accessible sources of information such as colleagues, medical representatives and drug companies, conferences and tertiary sources of information such as textbooks, and clinical practice guidelines (Furtado and Pereira, 2006; Layton et al., 2007; McGettigan et al., 2008; Oshikoya et al., 2011; Kosteniuk et al., 2013; Zielińska and Hermanowski, 2022), and online searchable databases (Almazrou et al., 2019; Qadus et al., 2022).

Using online sources to seek drug information is becoming increasingly prevalent among both healthcare providers and the general population (Brunetti and Hermes-DeSantis, 2010). However, due to the unsupervised nature of the Internet and inadequate regulation by many websites, not all of the information available online is accurate and not all websites are reliable (Brunetti and Hermes-DeSantis, 2010; Shields and Park, 2019). Therefore, healthcare providers should be aware of this and evaluate the information they get online and ensure that they only use credible and specialised websites.

Many research articles and studies have investigated the sources of drug information used by healthcare professionals and the challenges they face during this process (Furtado and Pereira, 2006; Layton et al., 2007; McGettigan et al., 2008; Tumwikirize et al., 2008; Oshikoya et al., 2011; Kosteniuk et al., 2013; Almazrou et al., 2019; Qadus et al., 2022; Zielińska and Hermanowski, 2022). However, as far as this research is aware, no studies have attempted to analyse the drug information-seeking behaviour of physicians in Jordan.

This research approached Jordanian physicians to evaluate their drug information needs, the sources of information they use and their perceived credibility, and the challenges they encounter when looking for accurate and contemporary information concerning drugs.

## 2 Methods and materials

### 2.1 Study design and population

This study is an observational, cross-sectional study conducted in Jordan throughout January and February 2023. The study’s protocol, design and questionnaire were officially approved by the Research Ethics Committee of the School of Medicine at Al-Balqa Applied University BAU (Reference Number: BMS/1/313, Proposal Number: 23/2022). The participants are practising physicians in Jordan and were selected regardless of their speciality, age, gender, seniority, or place of employment. Having established that the number of practising physicians registered at the Jordan Medical Association was approximately 42,000, a sample size of 380 was targeted to achieve a 5% margin of error and a confidence level of 95% assuming a null response distribution of 50%. The sample size was calculated using Raosoft^®^ (an online sampling calculator) (Sample Size Calculator by Raosoft and Inc, 2022). Participants were recruited using purposive sampling followed by snowball sampling. Participants were invited to fill out a self-administered questionnaire using either the online version that was created on Google Forms or the printed copy that was disseminated during clinic visits across the kingdom. The participants filled out and submitted the questionnaire anonymously and voluntarily.

### 2.2 Questionnaire

After a thorough literature review, a self-administered questionnaire was developed by the researchers. The first version of the questionnaire was validated (for both face and content validity were checked) by a panel of experts and a subsequent pilot run was conducted. When required, amendments were made, and the questionnaire was revalidated in its final version. The results from the pilot testing were excluded from the analysis.

The questionnaire (Supplementary Table S1) was written in Arabic, the official language of Jordan, and consisted of twenty-one multiple choice, checkboxes and Likert scale questions which were organised into multiple sections concerning the participants’ demographics and characteristics (11 questions), drug information that physicians require (one checkbox question with 14 different information type to choose from), the physicians’ information-seeking behaviour (one multiple choice question about the frequency of seeking drug information and 5 checkbox questions about the sources of information and their availability and one Likert scale question about drug information in doctors practice), challenges faced when looking for drug information (one Likert scale question with seven potential challenges to rate) and, finally, how the physicians perceived the reliability of the drug information sources (one Likert scale question with 10 drug information source to rate).

A cover letter was attached to the beginning of the questionnaire detailing the aims of the study, a confidentiality statement, and a voluntary participation statement. Participants were informed of their right to withdraw from the study at any time.

### 2.3 Statistical analysis

Data were analysed using IBM SPSS statistics (version 23) predictive analytics software. Categorical data were presented as frequencies and percentages and were compared using the Pearson Chi-square (χ2) test. *p*-values of <0.05 were considered statistically significant.

## 3 Results

A total of 380 physicians participated in the study, most of them were young (<30 years old) and the percentage of males was more than female (56.6% vs. 43.4%). Participants worked across all healthcare settings (hospitals, medical centres and clinics) with some of them working in more than one setting. Participants were, also, from all levels of seniority (interns, general practitioners, residents, and specialists). Most participants obtained their MD degree from Jordan (279, 73.4%) and studied in English (333, 87.6%). More than half of the participants (245, 64.5%) did not have access to the internet at their workplace (unless they use their own phones and internet connection) and (81, 21.3%) have worked in academia, Table 1.

**TABLE 1:** Demographics and general characteristics of the participants, *n* = 380.

	Frequency (%)
Gender	
Male	215 (56.6%)
Female	165 (43.4%)
Years of experience	
<10 years	291 (76.6%)
10–19 years	45 (11.8%)
20–29 years	22 (5.8%)
≥30 years	22 (5.8%)
Age	
<30 years	238 (62.6%)
30–39 years	75 (19.7%)
≥40 years	67 (17.6%)
Place of employment (practice setting)^a^	
Private hospital	110 (28.9%)
Governmental hospital	108 (28.4%)
Educational hospital	81 (21.3%)
Private clinic	69 (18.2%)
Royal medical services	40 (10.5%)
Healthcare centre	24 (6.3%)
Country of obtaining the medical degree	
Jordan	279 (73.4%)
North America	3 (0.8%)
Europe and United Kingdom	55 (14.5%)
Another Arab country (other than Jordan)	43 (11.3%)
Language of study	
English	333 (87.6%)
Arabic	7 (1.8%)
Language other than Arabic and English	40 (10.5%)
Seniority level	
Intern	110 (28.9%)
General practitioner	44 (11.6%)
Resident	126 (33.2%)
Specialist	100 (26.3%)
Currently working or have worked previously in academia	
No	299 (78.7%)
Yes	81 (21.3%)
Internet access at work (other than personal internet)	
No	245 (64.5%)
Yes	135 (35.5%)

^a^ Some participants reported working in more than one clinical setting.

### 3.1 Access to information sources

Most participants searched for drug information weekly (155, 40.8%), or daily (150, 39.5%), a few searched monthly (60, 15.8%), and a very limited number searched yearly or less (15, 3.9%). Dosage regimen (dose, frequency and duration) and adverse drug events were the most searched information among the participants, (342, 90.0%) and (315, 82.9%), respectively. Only (65, 17.1%) of the participants were offered access to subscription-only websites and journals by their workplace and almost half of the participants (189, 49.7%) tried to find the information they need from alternative, free, open-access websites and journals, Table 2.

**TABLE 2:** Access to information sources, *n* = 380.

	Frequency (%)
Frequency of accessing drug information	
Daily	150 (39.5%)
Weekly	155 (40.8%)
Monthly	60 (15.8%)
Yearly or less	15 (3.9%)
Type of drug information that physicians look for and access	
Dosage regimen (dose, frequency, and duration)	342 (90.0%)
Adverse drug events	315 (82.9%)
Dose adjustment in organ dysfunction (kidney, liver)	296 (77.9%)
Drug use during pregnancy and lactation	291 (76.6%)
Drug-drug and drug-food interactions	266 (70.0%)
Drug allergies	245 (64.5%)
Drug toxicity and management of overdose	221 (58.2%)
Mechanism of action	200 (52.6%)
Drugs monitoring and follow-up	185 (48.7%)
Drug prices	177 (46.6%)
Approved (label) use	146 (38.4%)
Off-label use	136 (35.8%)
Pill identification	110 (28.9%)
Ways to access and get information from subscription-only journals and/or websites	
I look for the same information in open-access sources	189 (49.7%)
I use Sci-Hub	121 (31.8%)
I pay for a subscription myself	84 (22.1%)
I use a colleague’s account	41 (10.8%)
I ask a colleague who has access to get me the information	38 (10%)
My workplace offers access to most websites and journals	41 (10.8%)
My workplace offers access to a few websites and journals	24 (6.3%)
Others^a^	48 (12.6%)

^a^ I do not need this type of information, or I get it from social groups.

### 3.2 Drug information sources and their perceived reliability

Physicians preferred to use online websites, textbooks and clinical practice guidelines to obtain drug information (327, 97.9%), (229, 60.3%) and (205, 60.0%) respectively. According to the participants’ opinion, the most reliable drug information sources were textbooks, clinical practice guidelines, and Pharmacopeia (US or British) respectively. Medical representatives were the least used and the least perceived reliable source of information by Jordanian physicians, Table 3.

**TABLE 3:** Drug sources used by physicians and their perceived reliability, *n* = 380.

	Responders who used the source n (%)	Responders who perceived the reliability n (%)		
		Low	Medium	High
Online websites	372 (97.9%)	13 (3.4%)	127 (33.4%)	240 (63.2%)
Clinical practice guidelines	229 (60.3%)	14 (3.7%)	52 (13.7%)	314 (82.6%)
Textbooks	228 (60.0%)	6 (1.6%)	28 (7.4%)	346 (91.1%)
Clinical research	205 (53.7%)	22 (5.8%)	69 (18.2%)	289 (76.1%)
Conferences, conventions, symposia, courses	168 (44.2%)	27 (7.1%)	104 (27.4%)	249 (65.5%)
Colleagues/Peers	167 (43.9%)	8 (2.1%)	108 (28.4%)	264 (69.5%)
Drug leaflet/Drug package insert	139 (36.6%)	N/A	N/A	N/A
Clinical pharmacists/Pharmacists	120 (31.6%)	10 (2.6%)	110 (28.9%)	260 (68.4%)
Pharmacopeia (US or British)	61 (16.1%)	18 (4.7%)	68 (17.9%)	294 (77.4%)
The National formulary	46 (12.1%)	26 (6.8%)	94 (24.7%)	260 (68.4%)
Medical representatives	37 (9.7%)	126 (33.2%)	219 (57.6%)	35 (9.2%)

**FIGURE 1:**
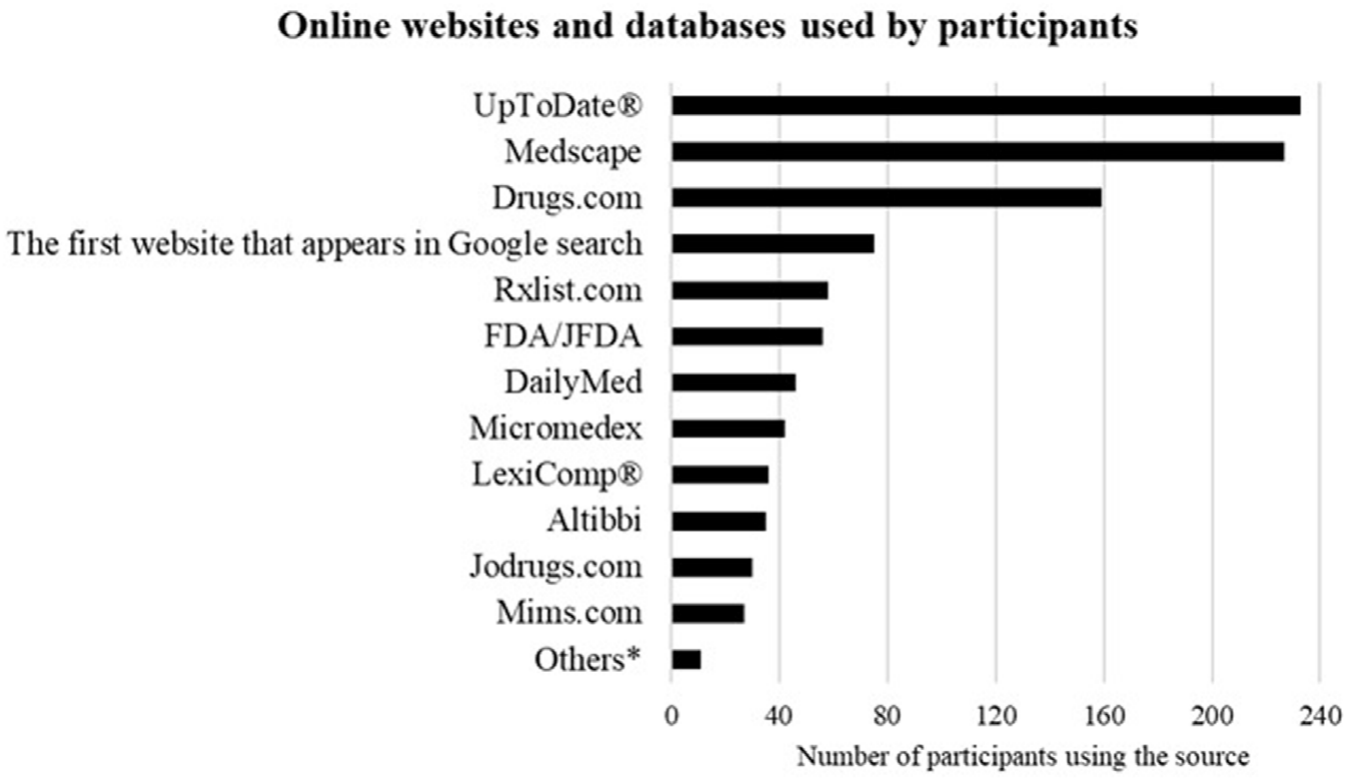
Websites that are used to get drug information. Participants (*n* = 380) were asked about the online websites and databases they use to get drug information. * Participants said that they use BMJ best practice, Amboss and Pubmed^®^.

The most commonly used websites for drug information were UpToDate^®^, Medscape, and Drugs.com (233, 61.3%), (227, 59.7%), and (159, 41.8%) respectively. Almost 20% of the participants use the first website that appears in a Google search, Figure 1.

### 3.3 Challenges that participants face and the factors affecting them

The most frequent challenge that physicians faced during their search for drug information was that they did not have access to subscription-only journals and websites (215, 56.6%), followed by difficulty knowing the trusted and credible source to use (15, 41.8%) and the huge number of sources available (134, 35.3%). Participants who work/worked in academia were less likely to face these challenges compared to participants who never worked in academia (*p* < 0.001), additionally, they were perceived as more able to interpret research results and understand statistics (*p* < 0.001), Table 3.

### 3.4 Drug information in participants’ daily practice

**FIGURE 2:**
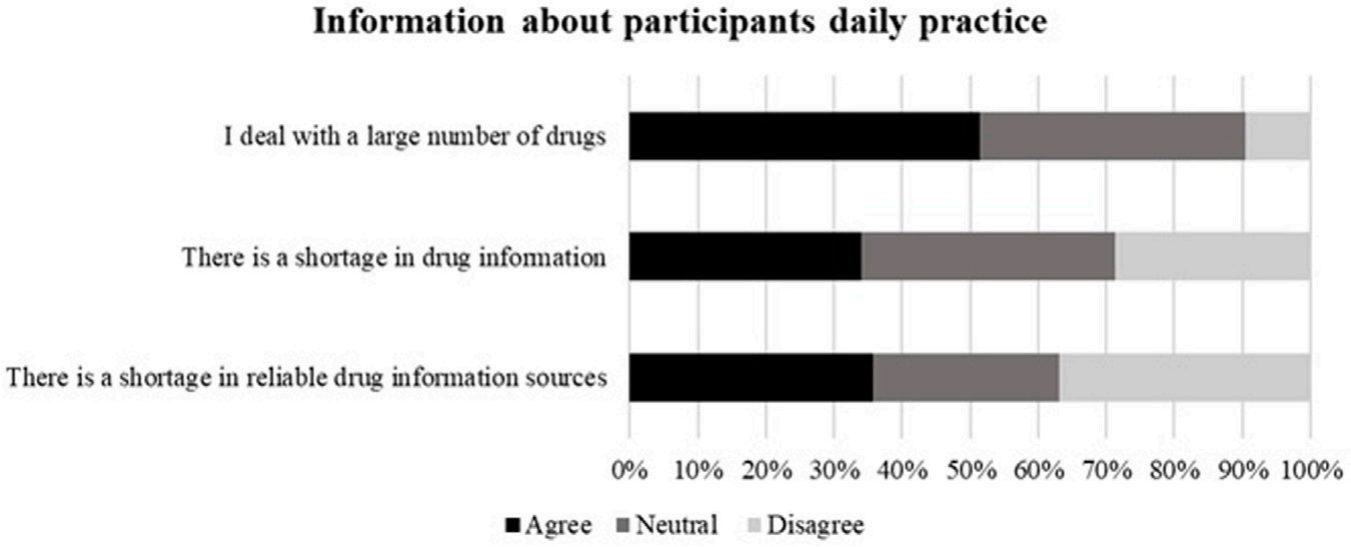
Information about participants’ daily practice. Participants (*n* = 380) were asked questions related to drugs and drug information related to their daily practice.

Half of the participants agreed that they deal with a large number of drugs (195, 51.3%) and one-third of participants agreed that there is a shortage of drug information and reliable sources to obtain this information in their practice, Figure 2.

When asked about what source of information they would recommend if their patients asked them for a reliable drug information source to know more about their drugs, most of the participants (137, 36.1%) said that they would provide all the necessary information to their patients. Sixty-one (16.1%) participants said that they would not recommend any source because it is dangerous for patients to follow recommendations without the physicians’ oversight. Fifty-five (14.5%) participants said that they would advise their patients to refer back to a pharmacist for more information on their drugs, 39 (10.3%) said that they would recommend reading the package insert, 6 (1.6%) would recommend Google, and 82 (21.6%) said that their recommendation of a source depends on the patient´s educational level. Table 4.

**TABLE 4:** Challenges that participants face when looking for drug information, *n* = 380.

	All	Work/worked in academia		
		No	Yes	*p* ^a^
Language barrier				0.126
Do not agree	322 (84.7%)	248 (82.9%)	74 (91.4%)	
Neutral	49 (12.9%)	44 (14.7%)	5 (6.2%)	
Agree	9 (2.4%)	7 (2.3%)	2 (2.5%)	
Difficulty using technology				0.225
Do not agree	325 (85.5%)	255 (85.3%)	70 (86.4%)	
Neutral	45 (11.8%)	38 (12.7%)	7 (8.6%)	
Agree	10 (2.6%)	6 (2.0%)	4 (4.9%)	
No time to look for information				0.141
Do not agree	108 (28.4%)	82 (27.4%)	26 (32.1%)	
Neutral	177 (46.6%)	147 (49.2%)	30 (37.0%)	
Agree	95 (25.0%)	70 (23.4%)	25 (30.9%)	
The huge number of sources available				<0.001
Do not agree	68 (17.9%)	41 (13.7%)	27 (33.3%)	
Neutral	178 (46.8%)	151 (50.5%)	27 (33.3%)	
Agree	134 (35.3%)	107 (35.8%)	27 (33.3%)	
Difficulty knowing the trusted and credible source to use				<0.001
Do not agree	81 (21.3%)	52 (17.4%)	29 (35.8%)	
Neutral	140 (36.8%)	114 (38.1%)	26 (32.1%)	
Agree	159 (41.8%)	133 (44.5%)	26 (32.1%)	
No access to subscription-only journals and websites				<0.001
Do not agree	61 (16.1%)	37 (12.4%)	24 (29.6%)	
Neutral	104 (27.4%)	79 (26.4%)	24 (30.9%)	
Agree	215 (56.6%)	183 (61.2%)	32 (39.5%)	
Inability to interpret research results and understand statistics				<0.001
Do not agree	195 (51.3%)	138 (46.2%)	57 (70.4%)	
Neutral	132 (34.7%)	115 (38.5%)	17 (21.0%)	
Agree	53 (13.9%)	46 (15.4%)	7 (8.6%)	

^a^ Pearson Chi-square (χ2) test.

## 4 Discussion

This is the first research to study drug information-seeking behaviour among physicians in Jordan. In this study, physicians from all levels of care, clinical settings, seniority levels, experience and speciality were recruited. The data demonstrate that over half of the physicians regularly encounter a large number of drugs in their practice and 80.3% frequently needed to access and search for drug information on a daily or weekly basis. Similarly, a study conducted in Poland revealed that most primary care physicians used drug information sources multiple times per day or week (Zielińska and Hermanowski, 2022). In another study, Lua and others indicated that physicians receive several drug-related enquiries from patients daily, highlighting the importance of readily accessible and accurate drug information for effective patient care (Lua et al., 2011).

Ninety per cent of the participants in this study state that they typically search for information regarding the dosage regimen of drugs (dose, frequency and duration of treatment) and 82% frequently require information concerning adverse drug events (ADEs). Moreover, a substantial proportion of the participants searched for information concerning dose adjustment in organ dysfunction and drug use during pregnancy and lactation. Several studies showed similar results (Theodorou et al., 2009; Lua et al., 2011; Hermes-desantis et al., 2021; Seidel et al., 2023). In two of these studies, however, the majority of searches concerned adverse drug events. Additionally, the results of this study reveal a high demand for information concerning drug-drug interactions (DDIs) with 70% of participants expressing the need for such information. Studies that focused on DDI information sources were published by Nabovati et al. (2017) and Bergk et al. (2004) who emphasised the importance of locating a reliable source of information about DDIs (Bergk et al., 2004; Nabovati et al., 2017). Despite the potential for reducing healthcare expenditure (Buddhism, 2016; Frati et al., 2017; Al Zoubi et al., 2022), and being an area of serious patient concern (Lua et al., 2011), less than half of the participants in this study search for drug prices. Therefore, doctors in Jordan should be encouraged to prioritize awareness of drug costs and consider the use of generic alternatives, whenever possible, to mitigate pharmaceutical costs.

Physicians in Jordan rely on a variety of sources to obtain drug information. Although the participants acknowledge the lack of reliability of some websites, almost all of the surveyed doctors (97.9%) reported using online websites which may be attributed to the speed of access, availability, and ease of use at the point of care. Clinical practice guidelines and textbooks have the highest perceived reliability and are used by 60.3% and 60.0% of the participants, respectively. Conversely, medical representatives are the least used and are identified as the least reliable source of information in the opinion of Jordanian doctors. Additionally, a significant number of doctors (167, 43.9%) seek information from their colleagues or peers. International studies contained results which were comparable and dissimilar to those contained in this research. A recent study, published in 2021, demonstrated that most healthcare professionals used the internet, either specialised websites or general search engines, very frequently (Hermes-desantis et al., 2021). In another study, published in 2022, Zielińska and others stated that most Polish primary care physicians utilise national medical portals and clinical practice guidelines which were recognised as being very reliable. In the same study, human sources of information such as colleagues and medical representatives were less frequently used (Zielińska and Hermanowski, 2022). Conversely, colleagues were identified as the primary source of information for doctors in public hospitals in Uganda (Tumwikirize et al., 2008) and educational hospitals in Nigeria (Oshikoya et al., 2011) while medical representatives were one of the main sources of information among Estonian physicians (Raal et al., 2006) and general practitioners in Australia (McGettigan et al., 2008). The utilisation of various sources of information can be justified as the selection of the information sources depends on the information needed, the user characteristics and the clinical setting.

While the majority of participants in this study reported using online websites to search for and obtain drug information, only 35.5% of the participants had internet access available at their workplace and even fewer (17.1%) have access to selected subscription-only journals and websites through their professional roles. Most participants use their smartphones to access the internet while some funded their subscriptions or sought alternative ways to obtain the required information. UpToDate^®^, Medscape and Drugs.com are the most frequently used databases by physicians in Jordan. Similarly, physicians in Saudi Arabia (Almazrou et al., 2019) and Singapore (Lua et al., 2011) use UpToDate^®^ as a reliable online source of information. Almost 20% of the physicians in this study use the first result shown in a Google search. In a study from Singapore, 50% of the participants use Google search to get drug information; however, most of them check the credibility of the websites or verify the information on other websites (Lua et al., 2011). While Google is favoured for its speed and user-friendly interface, it is essential to encourage healthcare providers to assess the reliability of the search results, as not all information available on the internet is organised or supervised.

Physicians face many challenges, difficulties and impediments when searching for drug information. Most drug information sources (online drug information databases, international peer-reviewed journals, clinical practice guidelines and textbooks) are available in English which can be a challenge to some non-English speaking professionals (Zielińska and Hermanowski, 2022). However, in the current study, the language barrier was not considered a challenge because more than 85% of participants obtained their MD qualification from universities that teach in English. The participants encountered no issues when using online sources which might be due to being part of a younger demographic, their familiarity with new technology, and their ability to adapt to technological advancements. Recent statistics by the Jordan Medical Association showed that more than half of Jordanian doctors are under the age of 40 (News, 2023). This can explain the high percentage of young participants in our study, making our sample representative of the target population. Additionally, younger doctors were easier to access and were more cooperative to participate in this study. Several studies note that a lack of time (Sharma et al., 2017; Campbell et al., 2021; Zielińska and Hermanowski, 2022) and limited access to credible drug information sources (Tumwikirize et al., 2008; Sharma et al., 2017; Zielińska and Hermanowski, 2022) are the main obstacles when searching for drug information; approximately a quarter of the physicians in this study reported that time constraints are a challenge. The most frequent challenges encountered concerned the vast number of available sources, knowing which sources are trusted and credible, and an inability to access subscription-only journals and websites, although these issues were less problematic for physicians who work, or have worked, in academia (*p* < 0.001). Academics exhibited greater confidence in interpreting research results and understanding statistics (*p* < 0.001) although it was not a big challenge to the rest of the participants which suggests that being in academia makes the process of information-seeking easier and emphasises the role of academics in transferring their knowledge and experience to their non-academic colleagues and the coming generations of physicians by organising workshops and lectures for physicians as a part of a continuous education scheme and incorporating new lectures and courses within the curricula of undergraduate MD programmes to enable the future generations of physicians to distinguish credible from non-credible information they find online and understand statistics and apprise literature critically and improve their time management skills.

The main strength of this study exists in its originality; it is the first study to describe the information-seeking behaviour of Jordanian doctors and the challenges they face throughout this process. Additionally, it draws attention to the important role academics can play in helping future doctors who encounter these challenges. On the other hand, there are a few challenges and limitations in this study. Despite reaching the target sample size (380 participants), a bigger number would have revealed the differences in information-seeking behaviours and challenges faced among minorities in this study such as physicians with rare specialties, physicians who studied their MD in languages other than English or older generations of physicians (60 years or older). However, reaching a bigger sample size was a huge challenge as many physicians were not able to participate in this study because of their busy schedules. Therefore, we recommend conducting future studies to focus on these groups and highlight their drug information-seeking behaviour and the challenges they face during this process.

## 5 Conclusion

This study revealed that Jordanian physicians are required to be knowledgeable about a large number of drugs in their daily practice and they are frequently required to search for and obtain drug information. The majority use online websites to obtain drug information such as dosage regimens and adverse drug events. Many doctors, specifically those with no prior experience working in academia, face challenges throughout this process due to factors such as the vast number of available sources, assessing the reliability of the information they have located, and accessing some sources. This suggests that being in academia makes the process of information-seeking easier which emphasises the crucial role played by academics who can transfer their knowledge and experience to their non-academic colleagues and future physicians which can be achieved via the organisation of training sessions and workshops and the provision of teaching materials to medical students throughout their study.

## Data Availability

The raw data supporting the conclusion of this article will be made available by the authors, without undue reservation.
