# Supplementary material for: Drug information-seeking behaviour among Jordanian physicians: a cross-sectional study
Source: Front Pharmacol. 2023 Nov 13;14:1264794. doi: 10.3389/fphar.2023.1264794 (PMC10679432; doi:10.3389/fphar.2023.1264794)
Supplement: Supplementary file 1 [file DataSheet1.PDF]

Supplementary Table 1. The validated questionnaire.

صممت هذه الدراسة لوصف استخدام مصادر الأدوية المختلفة وموثوقيتها ، والتحديات التي تواجه الأطباء في الأردن عند استخدامها. سوف يستغرق الأمر من 5 إلى 10 دقائق لملء هذا الاستبيان وبملئه فإنك تسمح لنا باستخدام المعلومات التي قدمتها. ستتم دراسة الاستبيانات دون الكشف عن هوية المشاركين وسيتم التعامل معها بسرية تامة.

	**المعلومات الديموغرافية وصفات المشاركين**							
1	**الجنس**	□ ذكر	□ أنثى					
2	**العمر**	□ أقل من ٣٠	□٣٠ –٣٩	□ ٤٠ – ٤٩	□ ٥٠ – ٥٩	□ ٦٠ أو أكثر		
3	**عدد سنوات الخبرة**	□ أقل من ١٠	□ ١٠ – ١٩	□ ٢٠ – ٢٩	□ ٣٠ أو أكثر			
4	**مكان العمل**	□ عيادة خاصة<br>□ مركز صحي خاص	□ مركز صحي حكومي<br>□ الخدمات الطبية الملكية	□ مستشفى خاص<br>□ مستشفى حكومي	□ مستشفى جامعي<br>□ غير ذلك ______			
5	**بلد الحصول على درجة البكالوريوس**	□ الأردن	□ دولة عربية أخرى	□ أوروبا	□ بريطانيا	□ أمريكا	□ كندا	□ غير ذلك:
6	**لغة التعليم في بلد الدراسة**	□ الانجليزية	□ العربية	□ غير ذلك: ________				
7	**عملت سابقًا/أو حاليًا في القطاع الأكاديمي**	□ نعم	□ لا					
8	**الصفة الوظيفية**	□ طالب امتياز	□ طبيب مقيم	□ طبيب عام	□ أخصائي / استشاري			
9	**الاختصاص**	□ الباطني<br>□ الأطفال<br>□ الجراحة العامة	□ النسائية والتوليد<br>□ الطب النفسي<br>□ أمراض الروماتيزم	□ جراحة الأنف والأذن والحنجرة<br>□ جراحة العظام<br>□ طب وجراحة العيون	□ طب الأسرة<br>□ الجلدية والتناسلية<br>□ غير ذلك: ____			
10	**الدولة المانحة لآخردرجة اختصاص لك**	□ الأردن	□ دولة عربية أخرى	□ أوروبا	□ بريطانيا	□ أمريكا	□ كندا	□غير ذلك:
11	**أحتاج للبحث عن المعلومات الدوائية:**	□ يوميًـا	□ أسبوعيًـا	□ شهريًا	□ سنويًا	□ أقل من ذلك		

	**المعلومات الدوائية التي يحتاجها الأطباء**		
12	**هل يمكنك الوصول إلى الإنترنت في مكان عملك (دون الاعتماد على إنترنت الهاتف المحمول)؟**	□ نعم	□ لا
13	**تبحث عادة عن المعلومات الآتية:**	**نعم**	**لا**
	الأعراض الجانبية للأدوية.	□	□
	أسعار الأدوية.	□	□
	تفاعلات الأدوية مع الغذاء ومع بعضها البعض.	□	□
	موانع استخدام الأدوية.	□	□
	نظام الجرعات (الجرعة، التكرار، ومدة العلاج).	□	□
	مراقبة الأدوية ومتابعتها.	□	□
	الاستخدام المُصادق عليه للدواء.	□	□
	آلية عمل الدواء.	□	□
	استخدام الدواء أثناء الحمل أو الرضاعة.	□	□
	حساسية الدواء.	□	□
	تعديل جرعة الدواء في خلل وظائف الأعضاء (الكلى والكبد).	□	□
	سمية الدواء (التعامل مع الجرعة الزائدة).	□	□
	التعرف على شكل حبة/قرص الدواء.	□	□
	الاستخدامات غير المصادق عليها/المصرح بها للدواء.	□	□

	**سلوكيات الأطباء في البحث عن المعلومات**				
14	**أي مما يلي قد تختار أو تفضل كمصدر للحصول على المعلومات الدوائية المختلفة؟ (يمكنك اختيار أكثر من خيار واحد)**				
	□ مواقع الإنترنت	□ الصيادلة وأخصائي الصيدلة السريرية	□ الكتيب الوطني للوصفات الدوائية		
	□ الزملاء	□ المؤتمرات، الندوات، الدورات	□ الإرشادات/التوصيات العالمية للممارسة السريرية		
	□ الأوراق البحثية	□ النشرة الدوائية Drug leaflet	□ دستور الأدوية والعقاقير (الأمريكي/البريطاني)		
	□ الكتب الطبية	□ مندوبي الدعاية الطبية	□ غير ذلك (حدد): __________		
15	**في حال استخدامك لمواقع الانترنت فأي من مواقع الويب الآتية تستخدمه حاليًا أو قمت باستخدامه سابقًا للبحث عن المعلومات الدوائية؟ (يمكنك اختيار أكثر من واحد)**				
	□ أنا لا أبحث في مواقع الإنترنت	□ UpToDate	□ DailyMed	□ FDA أو JFDA	Micromedex□
	□ أول موقع يظهر لي في جوجل	□ Medscape	□ Drugs.com	□ Rxlist.com	□ Jodrugs.com
	Clinicaltrials.gov□	□ Lexicomp	□ Altibbi.com	□ Mims.com	□ غير ذلك: _____

16 **كيف تحصل على المعلومات من المجلات و / أو مواقع الويب التي تتطلب اشتراكا مدفوعا لاستخدامها؟** (يمكنك اختيار أكثر من واحد)

□ يوفر مكان عملي الوصول إلى معظم مواقع الويب والمجلات.
□ يتيح مكان عملي الوصول إلى عدد قليل من المواقع والمجلات.
□ أنا أدفع مقابل الاشتراك الشخصي.
□ أطلب المساعدة للحصول على المعلومات من زميل لديه اشتراكٌ
□ أستخدم حساب أحد الزملاء المشتركين بهذه المجلات/المواقع.
□ أستخدم Sci-Hub
□ أبحث عن المعلومات نفسها في المصادر المجانية.
□ لست بحاجة إلى معلومات من المجلات / أو مواقع الويب التي تطلب اشتراكا مدفوعا
□ غير ذلك:

17 **إذا كنت بحاجة إلى إجراء حسابات (مثل جرعة الدواء، ووظائف الكلى، ومؤشر كتلة الجسم، الخ...) فإنك (يمكنك اختيار تقوم بذلك: أكثر من واحد)**

□ باستخدام الآلة الحاسبة.
□ باستخدام تطبيقات الهاتف المحمول.
□ أطلب مساعدة أحد الزملاء في ذلك.
□ أقوم بسؤال الصيدلي/ الصيدلاني السريري
□ أستخدم مواقع ويب متخصصة في ذلك
□ لا أحتاج إلى إجراء أي حسابات في تخصصي.
□ غير ذلك:

18 **إذا طلب منك مريض مصدرًا موثوقًا لمعلومات الأدوية لمعرفة المزيد عن أدويته:**

□ أعطي مرضاي جميع المعلومات التي يحتاجونها.
□ لا أوصي بأي مصدر تجنبًا للأخطاء التي قد تنتج من بحث المرضى عن المعلومات بأنفسهم دون استشارة الطبيب أو الرجوع إليه للتأكد من صحتها.
□ يعتمد على خلفية المريض التعليمية.
□ أطلب منهم الرجوع إلى الصيدلي / الصيدلاني السريري.
□ أطلب منهم البحث عن المعلومات التي يريدونها باستخدام جوجل.
□ أطلب منهم قراءة نشرة الدواء Drug leaflet
□ غير ذلك:

	من واقع ممارستك السريرية لتخصصك:	أوافق	محايد	لا أوافق
19	أتعامل مع عدد كبير من الأدوية.	□	□	□
	هناك نقص في المعلومات الدوائية.	□	□	□
	هناك نقص في المصادر الموثوقة والمرجعية للحصول على المعلومات الدوائية.	□	□	□

التحديات التي يواجهها الأطباء عند البحث عن المعلومات الدوائية

	فيما يلي التحديات التي تواجهها عند البحث عن المعلومات الدوائية:	أوافق	محايد	لا أوافق
20	حاجز اللغة.	□	□	□
	صعوبة استخدام التكنولوجيا.	□	□	□
	عدم وجود وقت كاف للبحث عن المعلومات.	□	□	□
	العدد الهائل من مصادر المعلومات المتوافرة.	□	□	□
	صعوبة تحديد المصدر الموثوق الذي يجب استخدامه.	□	□	□
	عدم توافر إمكانية الوصول للمجلات والمواقع الالكترونية التي تتطلب اشتراك مسبق بتكلفة عالية.	□	□	□
	صعوبة تحليل وفهم نتائج البحث والإحصائيات.	□	□	□

مدى موثوقية مصادر المعلومات الدوائية من وجهة نظر الأطباء

	برأيك، ما مدى موثوقية المصادر الآتية للمعلومات الدوائية:	لأقصى حد	كثيرًا	متوسط	قليلًا	على الإطلاق
21	مندوبي الأدوية.	□	□	□	□	□
	المؤتمرات، الندوات والدورات.	□	□	□	□	□
	الزملاء الأطباء.	□	□	□	□	□
	الصيادلة وأخصائي الصيدلة السريرية.	□	□	□	□	□
	مواقع الويب المتخصصة.	□	□	□	□	□
	الأوراق البحثية.	□	□	□	□	□
	الكتب الطبية.	□	□	□	□	□
	كتيب الوصفات الدوائية الوطني. The National Formulary	□	□	□	□	□
	إرشادات وتوصيات الممارسة السريرية Clinical Guidelines	□	□	□	□	□
	دستور الأدوية والعقاقير (الأمريكي/البريطاني)	□	□	□	□	□

شكرا على المشاركة وتعبئة الاستبيان. يرجى إعلامنا إذا كان لديك أي استفسار أو تعليق حول هذه الدراسة
